# Supplementary figures and images for: In vivo dendritic cell reprogramming for cancer immunotherapy
Source: Science. Author manuscript; Available in PMC 2024 Nov 1. (PMC7616765; doi:10.1126/science.adn9083)

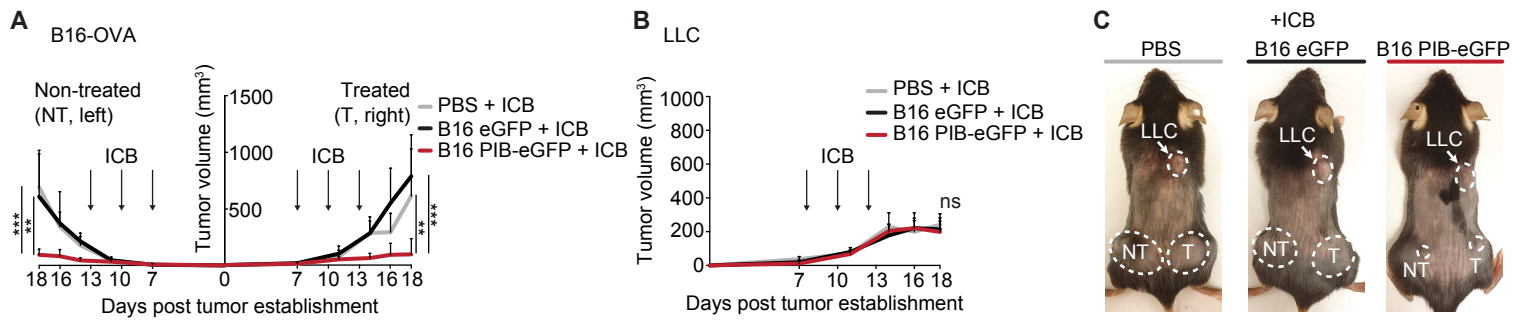

### Impact of TLR3 stimulation on cDC1-like cells in antitumor immunity

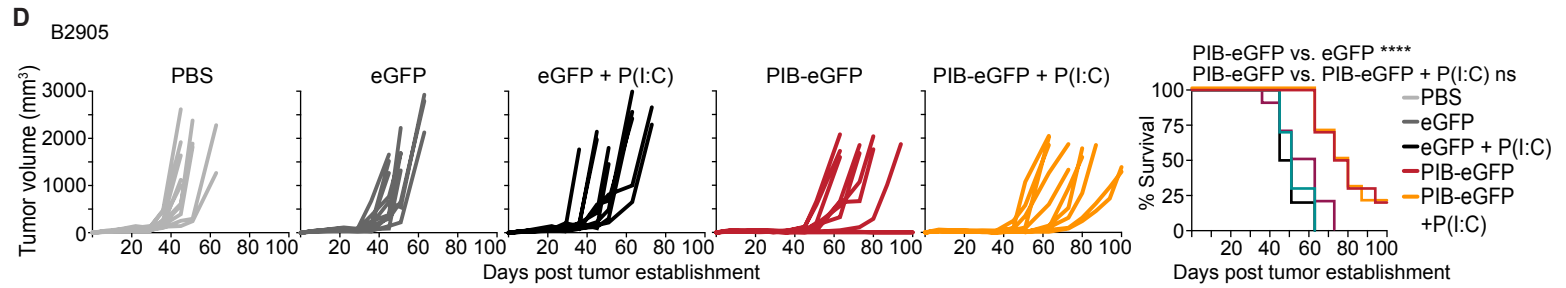

Supplement: Figure S1 [file EMS198548-supplement-Figure_S1.pdf]

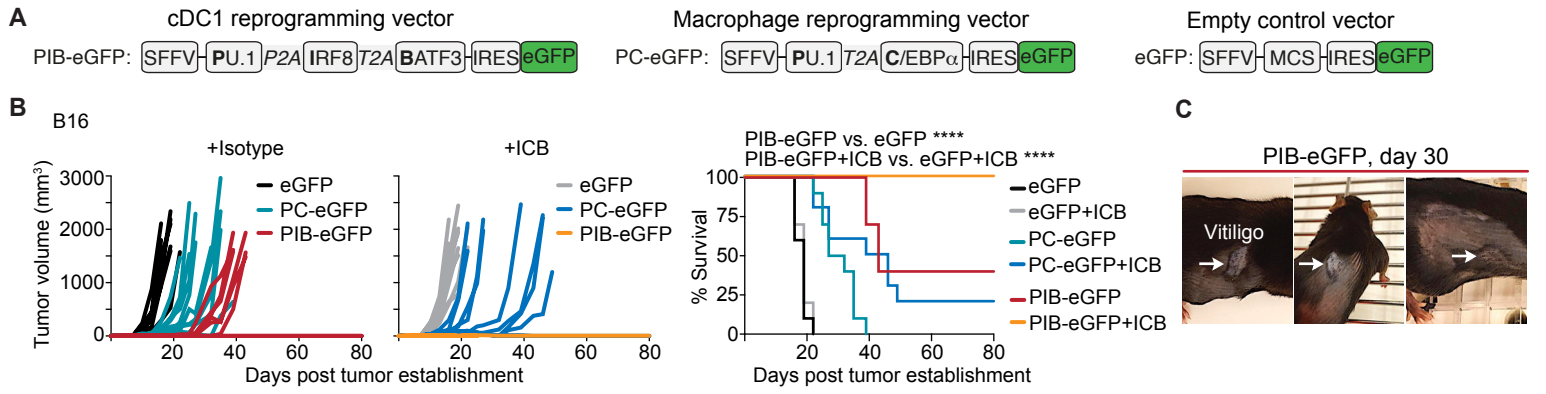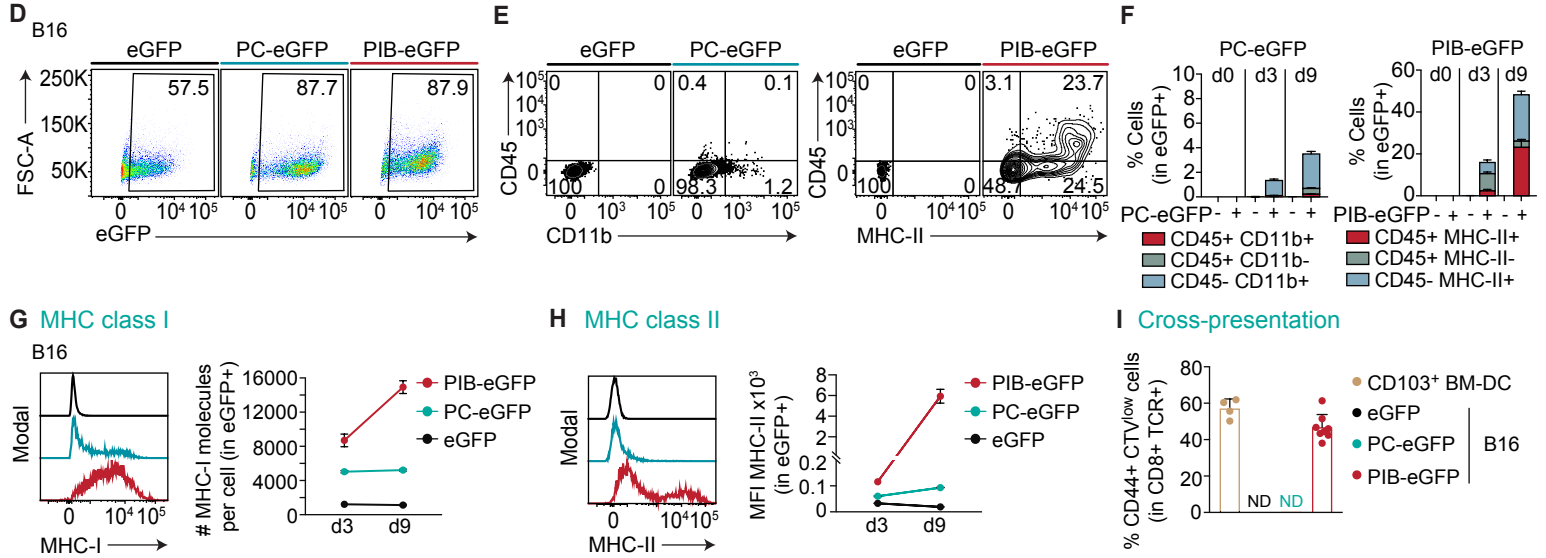

Supplement: Figure S2 [file EMS198548-supplement-Figure_S2.pdf]

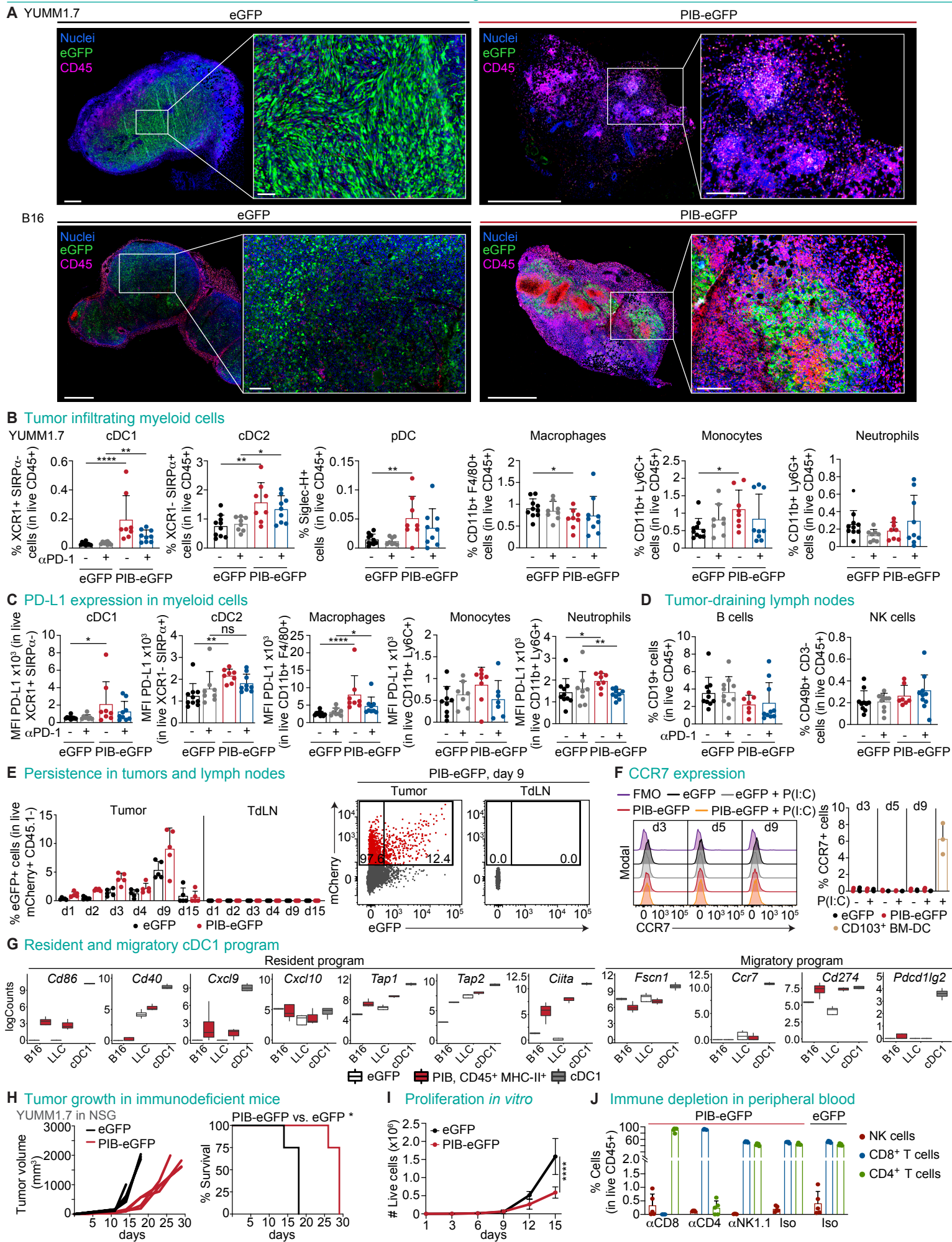

Supplement: Figure S4 [file EMS198548-supplement-Figure_S4.pdf]

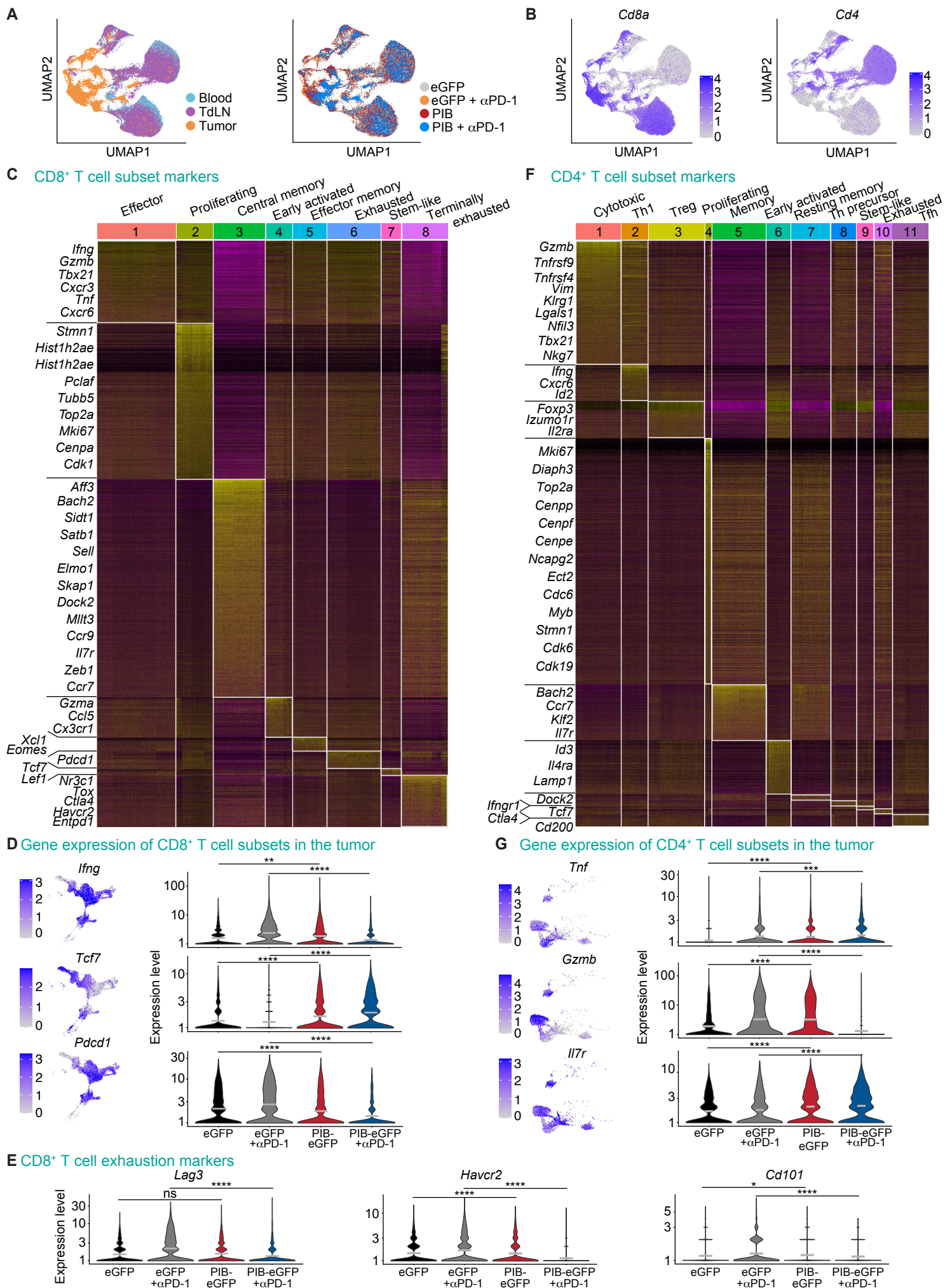

Supplement: Figure S5 [file EMS198548-supplement-Figure_S5.pdf]

**A Gene therapy safety**

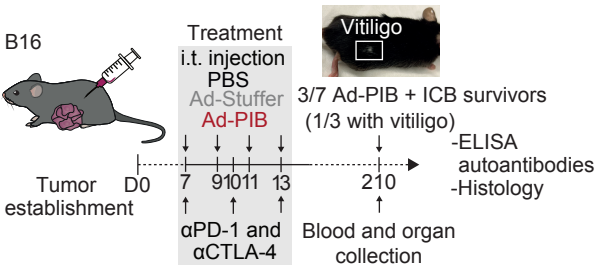

**B Autoantibodies**

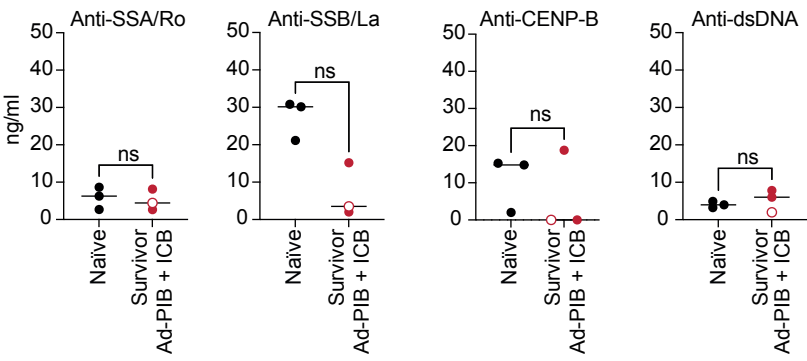

**C Histology**

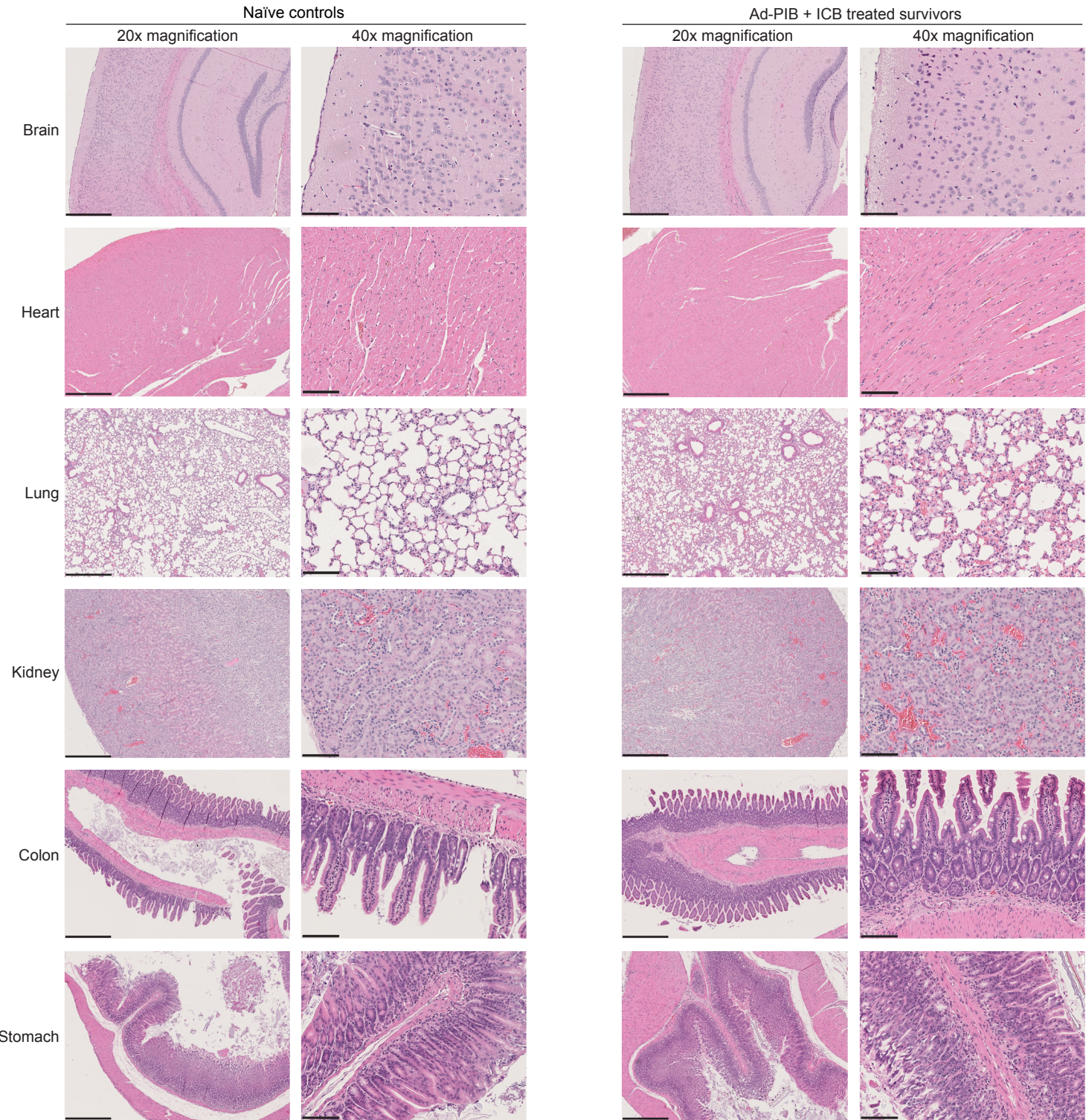

Supplement: Figure S12 [file EMS198548-supplement-Figure_S12.pdf]
