# Supplementary material for: In vivo dendritic cell reprogramming for cancer immunotherapy
Source: Science. Author manuscript; Available in PMC 2024 Nov 1. (PMC7616765; doi:10.1126/science.adn9083)
Supplement: Figure S3 [file EMS198548-supplement-Figure_S3.pdf]

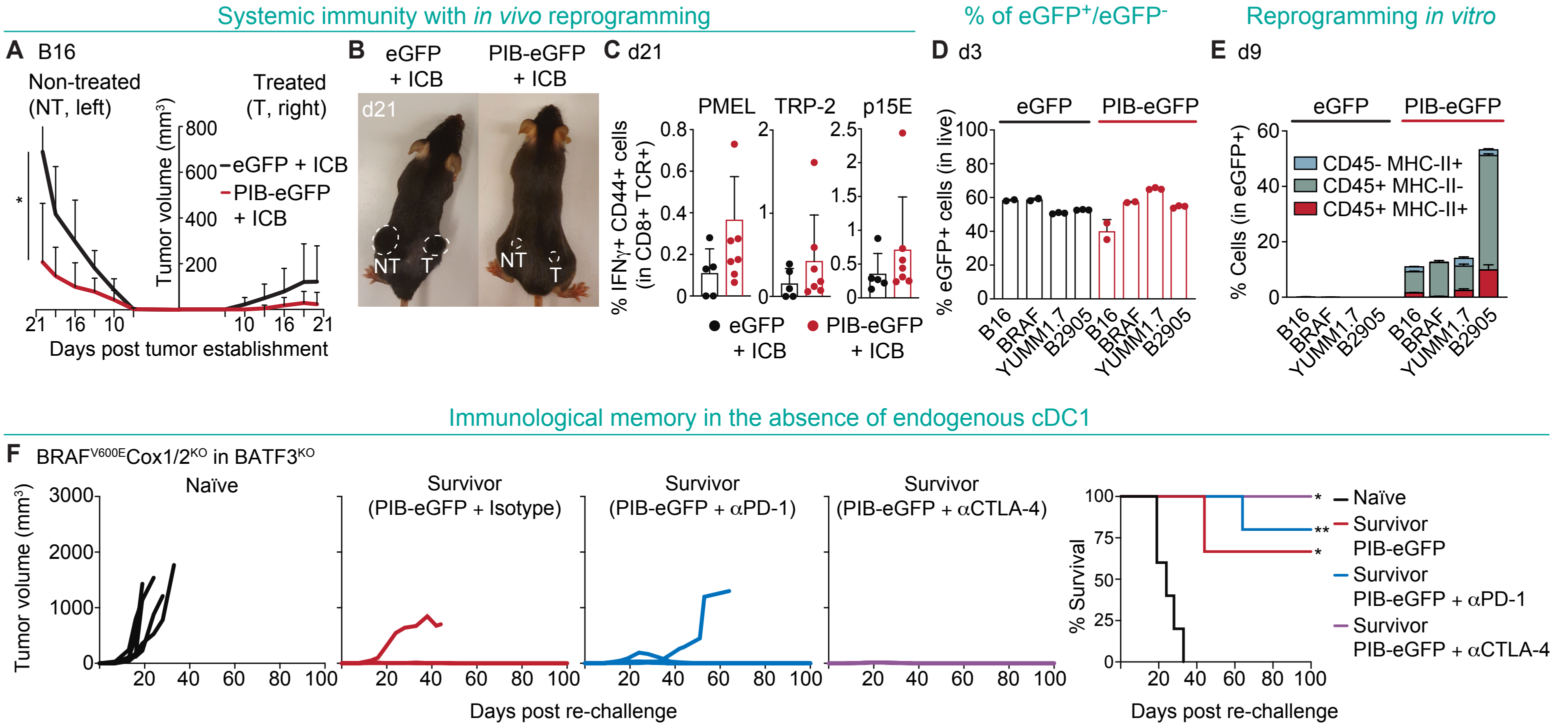

Systemic immunity with in vivo reprogramming
% of eGFP+/eGFP-
Reprogramming in vitro
A B16
Non-treated (NT, left)
Treated (T, right)
Tumor volume (mm3)
800
600
400
200
eGFP + ICB
PIB-eGFP + ICB
*
21 16 10
10 16 21
Days post tumor establishment
B eGFP + ICB
PIB-eGFP + ICB
d21
NT
T
C d21
PMEL
TRP-2
p15E
% IFNγ+ CD44+ cells (in CD8+ TCR+)
eGFP + ICB
PIB-eGFP + ICB
D d3
eGFP
PIB-eGFP
% eGFP+ cells (in live)
B16
BRAF
YUMM1.7
B2905
E d9
% Cells (in eGFP+)
CD45- MHC-II+
CD45+ MHC-II-
CD45+ MHC-II+
Immunological memory in the absence of endogenous cDC1
F BRAFV600ECox1/2KO in BATF3KO
Tumor volume (mm3)
Naïve
Survivor (PIB-eGFP + Isotype)
Survivor (PIB-eGFP + αPD-1)
Survivor (PIB-eGFP + αCTLA-4)
Days post re-challenge
% Survival
Naïve
Survivor PIB-eGFP
Survivor PIB-eGFP + αPD-1
Survivor PIB-eGFP + αCTLA-4
Days post re-challenge
