# Supplementary material for: In vivo dendritic cell reprogramming for cancer immunotherapy
Source: Science. Author manuscript; Available in PMC 2024 Nov 1. (PMC7616765; doi:10.1126/science.adn9083)
Supplement: Figure S6 [file EMS198548-supplement-Figure_S6.pdf]

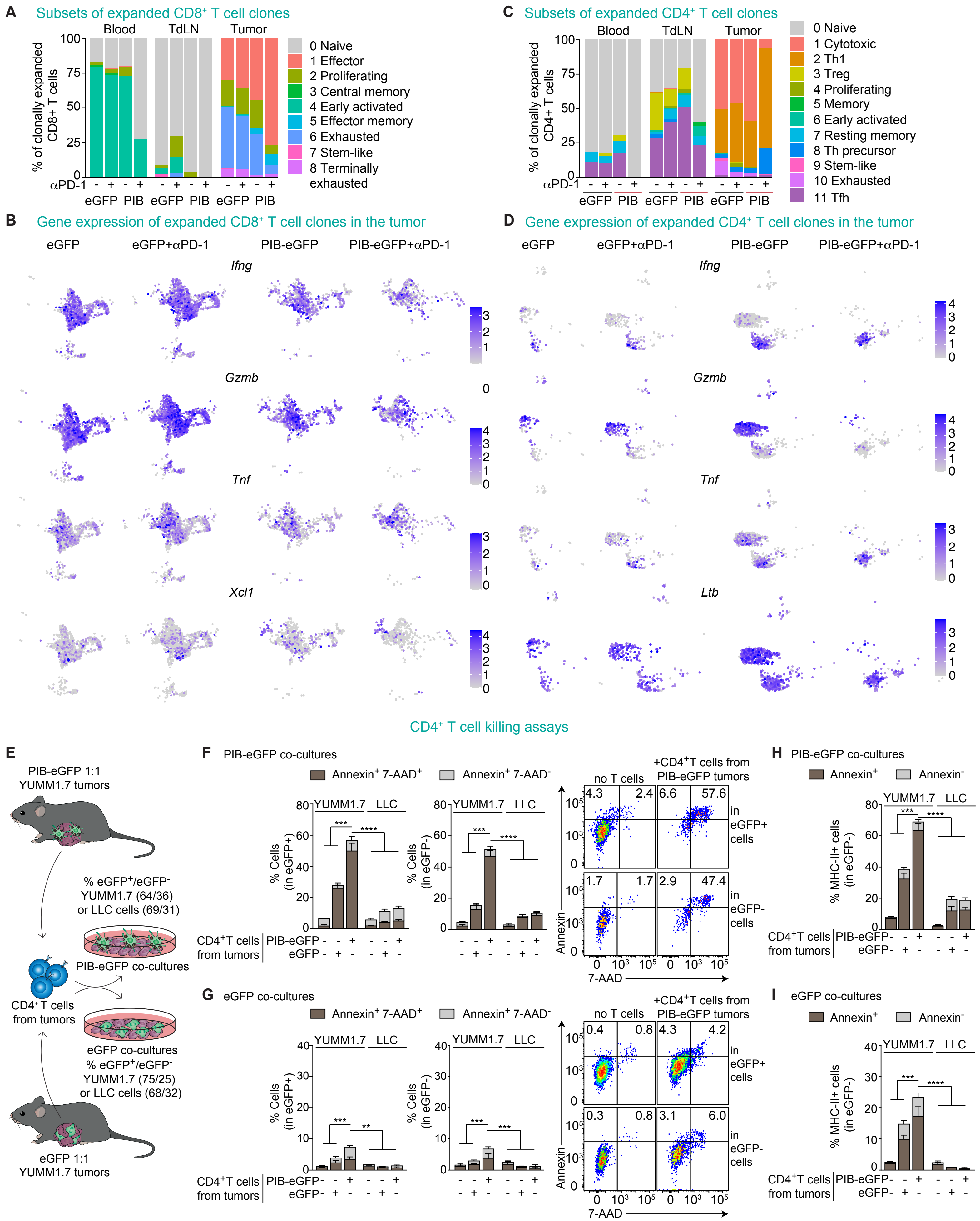
A
Subsets of expanded CD8+ T cell clones
Blood
TdLN
Tumor
% of clonally expanded CD8+ T cells
100
75
50
25
0
αPD-1
- + - +
eGFP PIB
0 Naive
1 Effector
2 Proliferating
3 Central memory
4 Early activated
5 Effector memory
6 Exhausted
7 Stem-like
8 Terminally exhausted
C
Subsets of expanded CD4+ T cell clones
% of clonally expanded CD4+ T cells
0 Naive
1 Cytotoxic
2 Th1
3 Treg
4 Proliferating
5 Memory
6 Early activated
7 Resting memory
8 Th precursor
9 Stem-like
10 Exhausted
11 Tfh
B
Gene expression of expanded CD8+ T cell clones in the tumor
eGFP
eGFP+αPD-1
PIB-eGFP
PIB-eGFP+αPD-1
Ifng
Gzmb
Tnf
Xcl1
D
Gene expression of expanded CD4+ T cell clones in the tumor
Ifng
Gzmb
Tnf
Ltb
CD4+ T cell killing assays
E
PIB-eGFP 1:1
YUMM1.7 tumors
% eGFP+/eGFP-
YUMM1.7 (64/36)
or LLC cells (69/31)
PIB-eGFP co-cultures
CD4+ T cells
from tumors
eGFP co-cultures
% eGFP+/eGFP-
YUMM1.7 (75/25)
or LLC cells (68/32)
eGFP 1:1
YUMM1.7 tumors
F
PIB-eGFP co-cultures
Annexin+ 7-AAD+
Annexin+ 7-AAD-
YUMM1.7
LLC
% Cells (in eGFP+)
% Cells (in eGFP-)
CD4+T cells from tumors
PIB-eGFP
eGFP
no T cells
+CD4+T cells from PIB-eGFP tumors
4.3
2.4
6.6
57.6
1.7
1.7
2.9
47.4
in eGFP+ cells
in eGFP- cells
Annexin
7-AAD
G
eGFP co-cultures
0.4
0.8
4.3
4.2
0.3
0.8
3.1
6.0
H
PIB-eGFP co-cultures
Annexin+
Annexin-
% MHC-II+ cells (in eGFP-)
I
eGFP co-cultures
