# Supplementary material for: In vivo dendritic cell reprogramming for cancer immunotherapy
Source: Science. Author manuscript; Available in PMC 2024 Nov 1. (PMC7616765; doi:10.1126/science.adn9083)
Supplement: Figure S7 [file EMS198548-supplement-Figure_S7.pdf]

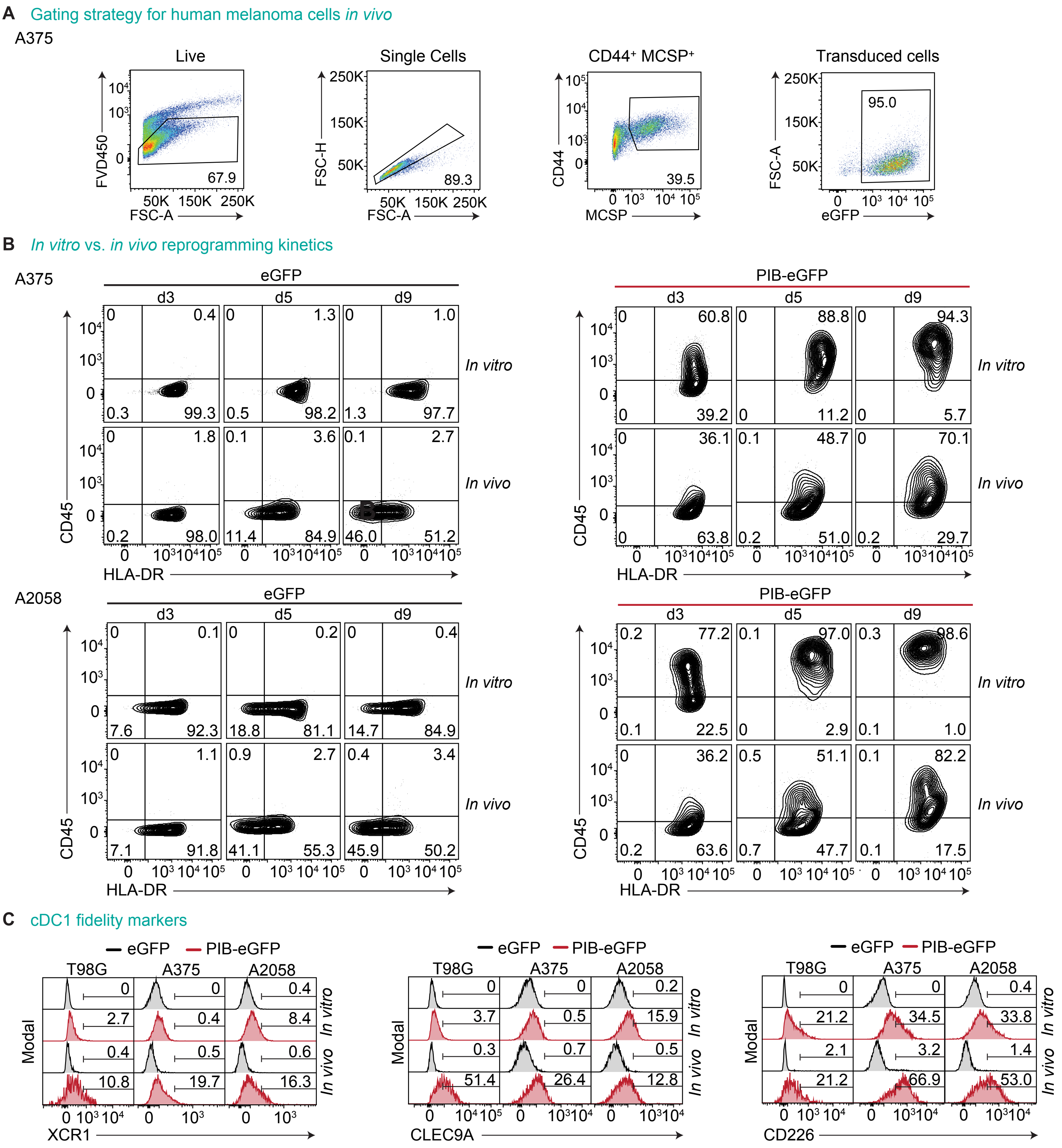
A Gating strategy for human melanoma cells in vivo
A375
Live
FVD450
FSC-A
67.9
Single Cells
FSC-H
FSC-A
89.3
CD44⁺ MCSP⁺
CD44
MCSP
39.5
Transduced cells
FSC-A
eGFP
95.0
B In vitro vs. in vivo reprogramming kinetics
A375
eGFP
PIB-eGFP
d3
d5
d9
In vitro
In vivo
CD45
HLA-DR
0 0.4 0.3 99.3
0 1.3 0.5 98.2
0 1.0 1.3 97.7
0 1.8 0.2 98.0
0.1 3.6 11.4 84.9
0.1 2.7 46.0 51.2
0 60.8 0 39.2
0 88.8 0 11.2
0 94.3 0 5.7
0 36.1 0 63.8
0.1 48.7 0.2 51.0
0 70.1 0.2 29.7
A2058
0 0.1 7.6 92.3
0 0.2 18.8 81.1
0 0.4 14.7 84.9
0 1.1 7.1 91.8
0.9 2.7 41.1 55.3
0.4 3.4 45.9 50.2
0.2 77.2 0.1 22.5
0.1 97.0 0 2.9
0.3 98.6 0.1 1.0
0 36.2 0.2 63.6
0.5 51.1 0.7 47.7
0.1 82.2 0.1 17.5
C cDC1 fidelity markers
eGFP
PIB-eGFP
T98G
A375
A2058
Modal
In vitro
In vivo
XCR1
0 0 0.4
2.7 0.4 8.4
0.4 0.5 0.6
10.8 19.7 16.3
CLEC9A
0 0 0.2
3.7 0.5 15.9
0.3 0.7 0.5
51.4 26.4 12.8
CD226
0 0 0.4
21.2 34.5 33.8
2.1 3.2 1.4
21.2 66.9 53.0
