# Supplementary material for: In vivo dendritic cell reprogramming for cancer immunotherapy
Source: Science. Author manuscript; Available in PMC 2024 Nov 1. (PMC7616765; doi:10.1126/science.adn9083)
Supplement: Figure S8 [file EMS198548-supplement-Figure_S8.pdf]

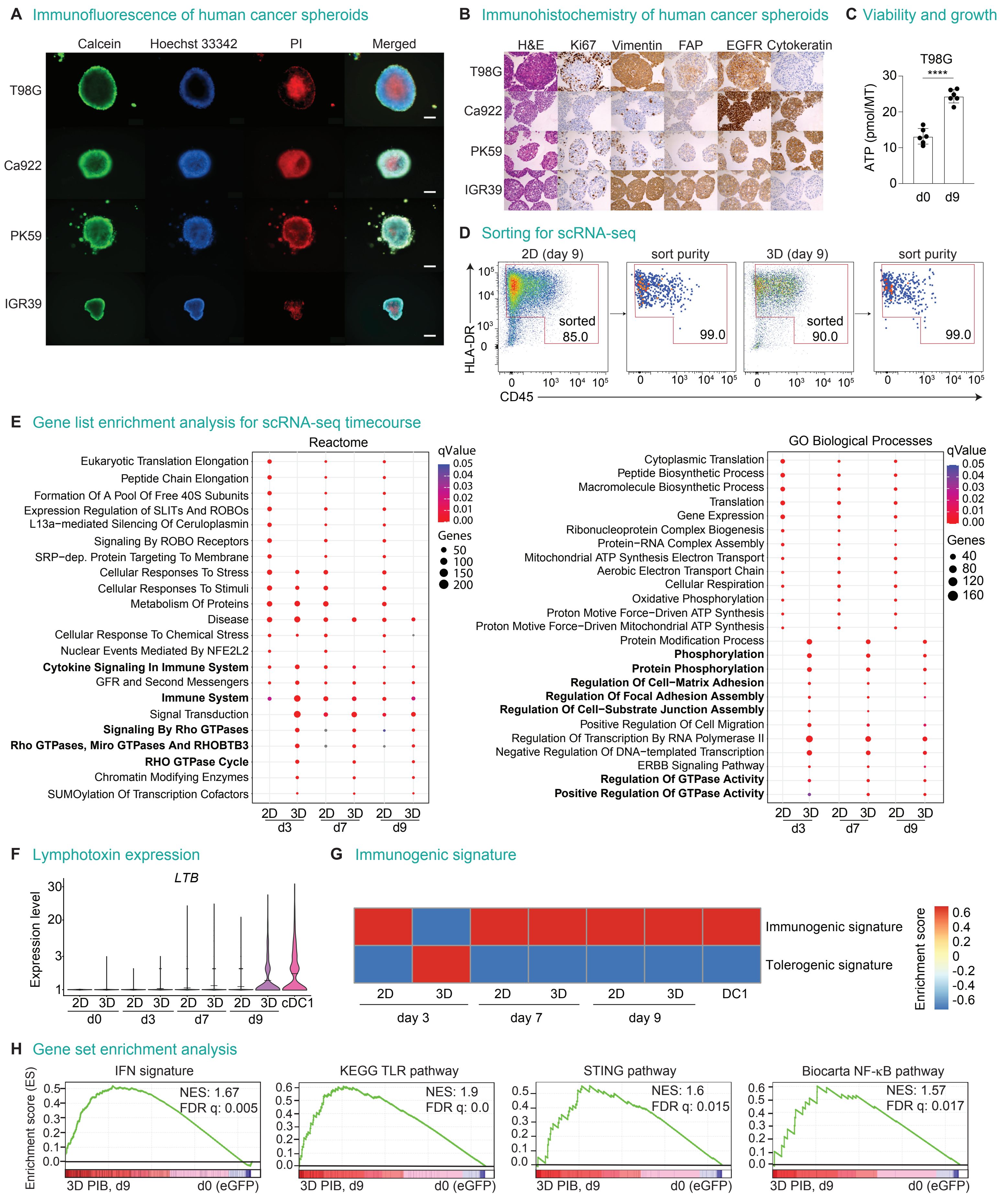
A Immunofluorescence of human cancer spheroids
Calcein
Hoechst 33342
PI
Merged
T98G
Ca922
PK59
IGR39
B Immunohistochemistry of human cancer spheroids
H&E
Ki67
Vimentin
FAP
EGFR
Cytokeratin
T98G
Ca922
PK59
IGR39
C Viability and growth
T98G
****
ATP (pmol/MT)
30
20
10
0
d0
d9
D Sorting for scRNA-seq
2D (day 9)
sort purity
3D (day 9)
sort purity
sorted 85.0
99.0
sorted 90.0
99.0
HLA-DR
CD45
E Gene list enrichment analysis for scRNA-seq timecourse
Reactome
Eukaryotic Translation Elongation
Peptide Chain Elongation
Formation Of A Pool Of Free 40S Subunits
Expression Regulation of SLITs And ROBOs
L13a−mediated Silencing Of Ceruloplasmin
Signaling By ROBO Receptors
SRP−dep. Protein Targeting To Membrane
Cellular Responses To Stress
Cellular Responses To Stimuli
Metabolism Of Proteins
Disease
Cellular Response To Chemical Stress
Nuclear Events Mediated By NFE2L2
Cytokine Signaling In Immune System
GFR and Second Messengers
Immune System
Signal Transduction
Signaling By Rho GTPases
Rho GTPases, Miro GTPases And RHOBTB3
RHO GTPase Cycle
Chromatin Modifying Enzymes
SUMOylation Of Transcription Cofactors
qValue
0.05
0.04
0.03
0.02
0.01
0.00
Genes
50
100
150
200
2D 3D 2D 3D 2D 3D
d3 d7 d9
GO Biological Processes
Cytoplasmic Translation
Peptide Biosynthetic Process
Macromolecule Biosynthetic Process
Translation
Gene Expression
Ribonucleoprotein Complex Biogenesis
Protein−RNA Complex Assembly
Mitochondrial ATP Synthesis Electron Transport
Aerobic Electron Transport Chain
Cellular Respiration
Oxidative Phosphorylation
Proton Motive Force−Driven ATP Synthesis
Proton Motive Force−Driven Mitochondrial ATP Synthesis
Protein Modification Process
Phosphorylation
Protein Phosphorylation
Regulation Of Cell−Matrix Adhesion
Regulation Of Focal Adhesion Assembly
Regulation Of Cell−Substrate Junction Assembly
Positive Regulation Of Cell Migration
Regulation Of Transcription By RNA Polymerase II
Negative Regulation Of DNA−templated Transcription
ERBB Signaling Pathway
Regulation Of GTPase Activity
Positive Regulation Of GTPase Activity
qValue
0.05
0.04
0.03
0.02
0.01
0.00
Genes
40
80
120
160
2D 3D 2D 3D 2D 3D
d3 d7 d9
F Lymphotoxin expression
LTB
Expression level
2D 3D 2D 3D 2D 3D 2D 3D cDC1
d0 d3 d7 d9
G Immunogenic signature
Immunogenic signature
Tolerogenic signature
2D 3D 2D 3D 2D 3D DC1
day 3 day 7 day 9
Enrichment score
H Gene set enrichment analysis
IFN signature
NES: 1.67
FDR q: 0.005
KEGG TLR pathway
NES: 1.9
FDR q: 0.0
STING pathway
NES: 1.6
FDR q: 0.015
Biocarta NF-κB pathway
NES: 1.57
FDR q: 0.017
Enrichment score (ES)
3D PIB, d9
d0 (eGFP)
