# Supplementary material for: In vivo dendritic cell reprogramming for cancer immunotherapy
Source: Science. Author manuscript; Available in PMC 2024 Nov 1. (PMC7616765; doi:10.1126/science.adn9083)
Supplement: Figure S9 [file EMS198548-supplement-Figure_S9.pdf]

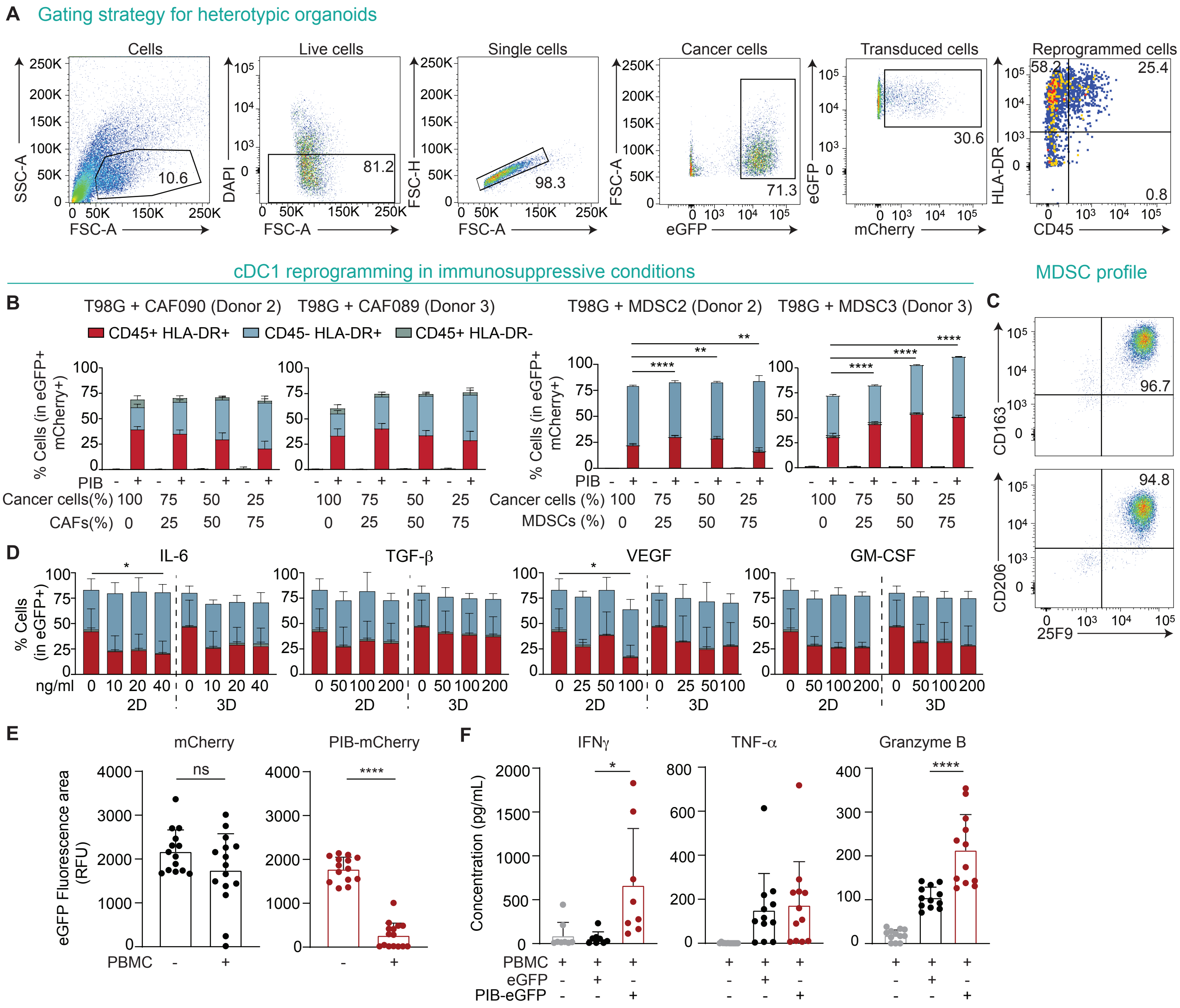

A Gating strategy for heterotypic organoids
Cells
Live cells
Single cells
Cancer cells
Transduced cells
Reprogrammed cells
SSC-A
FSC-A
DAPI
FSC-H
eGFP
mCherry
HLA-DR
CD45
10.6
81.2
98.3
71.3
30.6
58.2
25.4
0.8
cDC1 reprogramming in immunosuppressive conditions
MDSC profile
B
T98G + CAF090 (Donor 2)
T98G + CAF089 (Donor 3)
T98G + MDSC2 (Donor 2)
T98G + MDSC3 (Donor 3)
CD45+ HLA-DR+
CD45- HLA-DR+
CD45+ HLA-DR-
% Cells (in eGFP+ mCherry+)
PIB
Cancer cells(%) 100 75 50 25
CAFs(%) 0 25 50 75
Cancer cells (%) 100 75 50 25
MDSCs (%) 0 25 50 75
C
CD163
CD206
25F9
96.7
94.8
D
IL-6
TGF-β
VEGF
GM-CSF
% Cells (in eGFP+)
ng/ml
2D
3D
E
mCherry
PIB-mCherry
eGFP Fluorescence area (RFU)
PBMC
ns
F
IFNγ
TNF-α
Granzyme B
Concentration (pg/mL)
PBMC
eGFP
PIB-eGFP
