# Supplementary material for: In vivo dendritic cell reprogramming for cancer immunotherapy
Source: Science. Author manuscript; Available in PMC 2024 Nov 1. (PMC7616765; doi:10.1126/science.adn9083)
Supplement: Figure S10 [file EMS198548-supplement-Figure_S10.pdf]

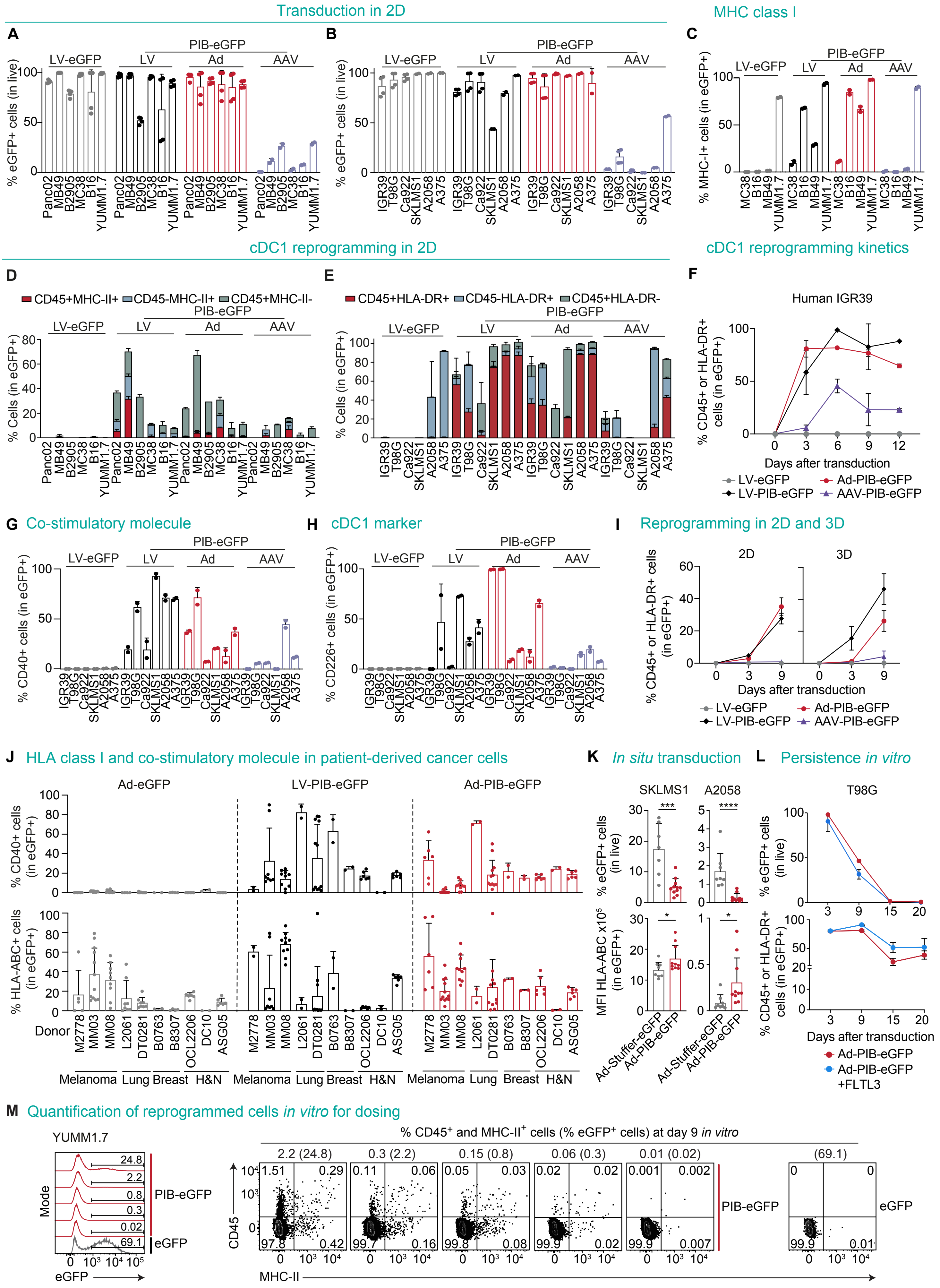
Transduction in 2D
MHC class I
A
B
C
LV-eGFP
PIB-eGFP
LV
Ad
AAV
% eGFP+ cells (in live)
Panc02
MB49
B2905
MC38
B16
YUMM1.7
IGR39
T98G
Ca922
SKLMS1
A2058
A375
% MHC-I+ cells (in eGFP+)
cDC1 reprogramming in 2D
cDC1 reprogramming kinetics
D
E
F
CD45+MHC-II+
CD45-MHC-II+
CD45+MHC-II-
CD45+HLA-DR+
CD45-HLA-DR+
CD45+HLA-DR-
% Cells (in eGFP+)
Human IGR39
% CD45+ or HLA-DR+ cells (in eGFP+)
Days after transduction
LV-eGFP
LV-PIB-eGFP
Ad-PIB-eGFP
AAV-PIB-eGFP
G
Co-stimulatory molecule
H
cDC1 marker
I
Reprogramming in 2D and 3D
% CD40+ cells (in eGFP+)
% CD226+ cells (in eGFP+)
2D
3D
% CD45+ or HLA-DR+ cells (in eGFP+)
J
HLA class I and co-stimulatory molecule in patient-derived cancer cells
K
In situ transduction
L
Persistence in vitro
Ad-eGFP
LV-PIB-eGFP
Ad-PIB-eGFP
% CD40+ cells (in eGFP+)
% HLA-ABC+ cells (in eGFP+)
Donor
M2778
MM03
MM08
L2061
DT0281
B0763
B8307
OCL2206
DC10
ASG05
Melanoma
Lung
Breast
H&N
SKLMS1
A2058
***
****
*
% eGFP+ cells (in live)
MFI HLA-ABC x10^5 (in eGFP+)
Ad-Stuffer-eGFP
Ad-PIB-eGFP
T98G
% eGFP+ cells (in live)
% CD45+ or HLA-DR+ cells (in eGFP+)
Days after transduction
Ad-PIB-eGFP
Ad-PIB-eGFP +FLTL3
M
Quantification of reprogrammed cells in vitro for dosing
YUMM1.7
Mode
24.8
2.2
0.8
0.3
0.02
69.1
PIB-eGFP
eGFP
eGFP
% CD45+ and MHC-II+ cells (% eGFP+ cells) at day 9 in vitro
2.2 (24.8)
0.3 (2.2)
0.15 (0.8)
0.06 (0.3)
0.01 (0.02)
(69.1)
1.51
0.29
97.8
0.42
0.11
0.06
99.7
0.16
0.05
0.03
99.8
0.08
0.02
0.02
99.9
0.02
0.001
0.002
99.9
0.007
0
0
99.9
0.01
CD45
MHC-II
PIB-eGFP
eGFP
