# Supplementary material for: In vivo dendritic cell reprogramming for cancer immunotherapy
Source: Science. Author manuscript; Available in PMC 2024 Nov 1. (PMC7616765; doi:10.1126/science.adn9083)
Supplement: Figure S11 [file EMS198548-supplement-Figure_S11.pdf]

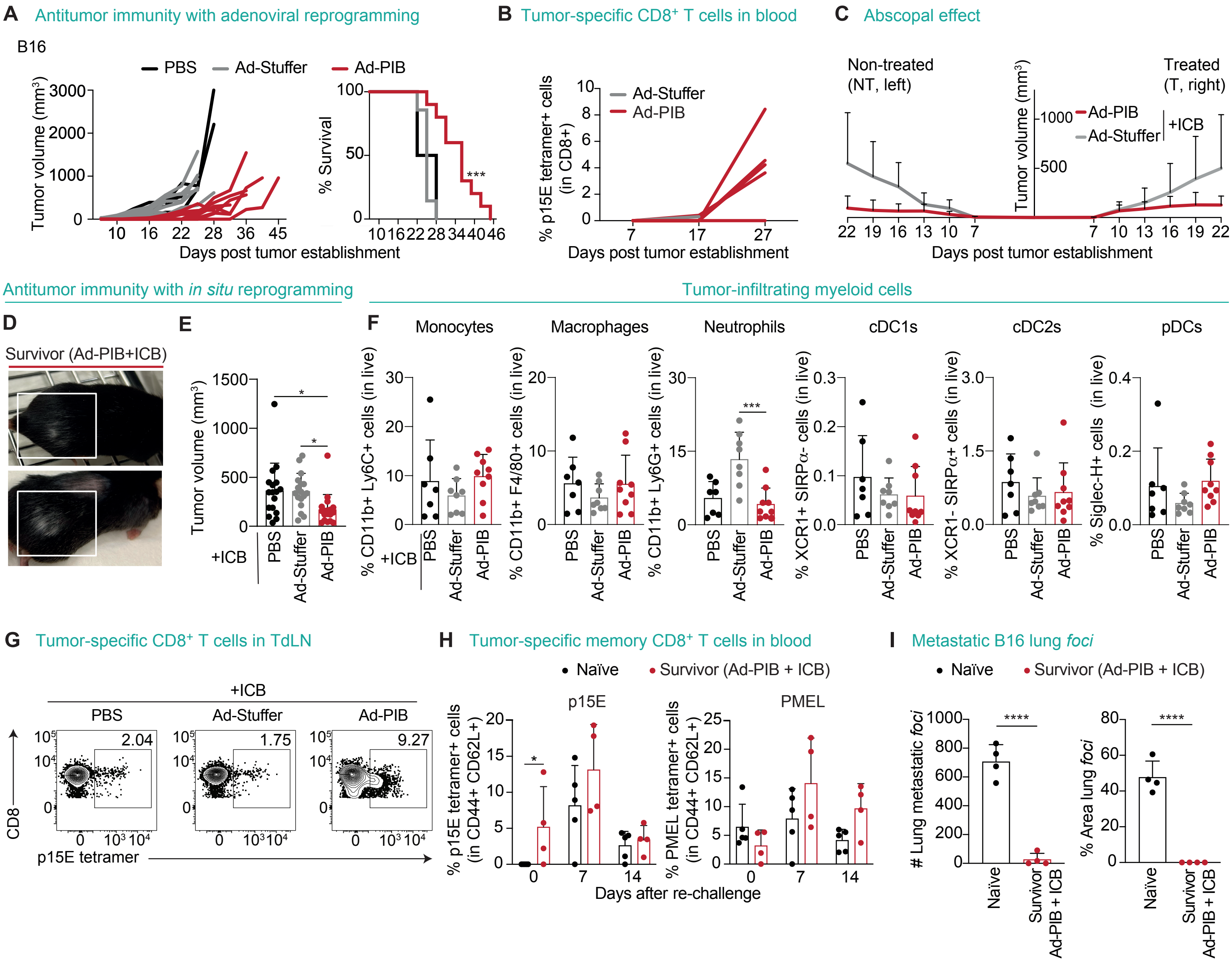
A Antitumor immunity with adenoviral reprogramming
B16
PBS
Ad-Stuffer
Ad-PIB
Tumor volume (mm³)
% Survival
***
Days post tumor establishment
B Tumor-specific CD8⁺ T cells in blood
% p15E tetramer+ cells (in CD8+)
Ad-Stuffer
Ad-PIB
Days post tumor establishment
C Abscopal effect
Non-treated (NT, left)
Treated (T, right)
Tumor volume (mm³)
Ad-PIB
Ad-Stuffer
+ICB
Days post tumor establishment
Antitumor immunity with in situ reprogramming
Tumor-infiltrating myeloid cells
D
Survivor (Ad-PIB+ICB)
E
Tumor volume (mm³)
+ICB
PBS
Ad-Stuffer
Ad-PIB
F
Monocytes
Macrophages
Neutrophils
cDC1s
cDC2s
pDCs
% CD11b+ Ly6C+ cells (in live)
% CD11b+ F4/80+ cells (in live)
% CD11b+ Ly6G+ cells (in live)
% XCR1+ SIRPα- cells (in live)
% XCR1- SIRPα+ cells (in live)
% Siglec-H+ cells (in live)
G Tumor-specific CD8⁺ T cells in TdLN
+ICB
PBS
Ad-Stuffer
Ad-PIB
2.04
1.75
9.27
CD8
p15E tetramer
H Tumor-specific memory CD8⁺ T cells in blood
Naïve
Survivor (Ad-PIB + ICB)
p15E
PMEL
% p15E tetramer+ cells (in CD44+ CD62L+)
% PMEL tetramer+ cells (in CD44+ CD62L+)
Days after re-challenge
I Metastatic B16 lung foci
Naïve
Survivor (Ad-PIB + ICB)
# Lung metastatic foci
% Area lung foci
****
Naïve
Survivor Ad-PIB + ICB
